# Model-informed precision dosing of vancomycin in clinical practice: an intervention development study

**DOI:** 10.1007/s11096-024-01822-x

**Published:** 2024-11-08

**Authors:** Maria Swartling, Anna-Karin Hamberg, Mia Furebring, Thomas Tängdén, Elisabet I. Nielsen

**Affiliations:** 1 Department of Pharmacy, Uppsala University, Box 580, 751 23 Uppsala, Sweden; 2 Department of Clinical Chemistry and Pharmacology, Uppsala University Hospital, Uppsala, Sweden; 3 Department of Medical Sciences, Infection Medicine, Uppsala University, Uppsala, Sweden

**Keywords:** Health plan implementation, Precision dosing, Precision medicine, Therapeutic drug monitoring, Vancomycin

## Abstract

**Background:**

Current guidelines recommend dosing vancomycin based on the area under the concentration time curve (AUC) to maximise efficacy and minimise the risk of nephrotoxicity. The preferred approach to AUC-guided therapy is to apply model-informed precision dosing (MIPD). However, the adoption in clinical practice has been slow.

**Aim:**

We aimed to develop an intervention, including a standardised MIPD workflow and an implementation plan for vancomycin AUC-guided dosing, in a Swedish tertiary hospital.

**Method:**

The intervention was developed in a framework-guided process. The design phase included stakeholder feedback (nurses, pharmacists, physicians), local data collection and feasibility testing of intervention components with parallel consideration of implementation aspects. The hypothesised relationships between the different components, implementation strategies and the mechanism of action resulting in expected outcomes were represented by a logic model.

**Results:**

The final intervention consisted of a workflow for MIPD, with defined roles and responsibilities, as well as processes for data and information transfer. Details were provided in supportive documents; an instruction on therapeutic drug monitoring (TDM) sampling and documentation for nurses, and a detailed dosing software instruction for MIPD consultants and clinical pharmacists. Activities to facilitate implementation included the development of a local clinical routine for vancomycin dosing, staff training and recurring MIPD rounds.

**Conclusion:**

An intervention for MIPD, with an implementation plan for AUC-guided dosing of vancomycin, was developed for a tertiary hospital setting. The process can be used as guidance for other institutions with similar context wishing to initiate MIPD.

**Supplementary Information:**

The online version contains supplementary material available at 10.1007/s11096-024-01822-x.

## Impact statements

- The developed workflow provides a platform solution for model-informed precision dosing (MIPD) that can be applied for various drugs, to support the tailoring of doses to patients’ needs.
- The work introduces a new MIPD expert role, and new responsibilities for clinical pharmacists at Swedish hospitals, providing an opportunity for professional development.

## Introduction

Vancomycin is used in the treatment of infections caused by Gram-positive bacteria, such as methicillin-resistant *Staphylococcus aureus* (MRSA) infections. The effect of vancomycin has been shown to correlate to the ratio of the area under the plasma concentration time curve (AUC) to the minimum inhibitory concentration (MIC) [1–4]. Vancomycin-associated nephrotoxicity is a concern with reported prevalence 5–43% [5]. Therapeutic drug monitoring (TDM) is often used to guide vancomycin therapy to increase the likelihood of therapeutic yet non-toxic drug exposures [6]. International evidence-based guidelines from 2020 [7] recommend dosing vancomycin based on the total exposure, i.e., AUC, instead of the previously recommended trough concentrations. This is supported by a meta-analysis showing that an AUC-guided approach results in lower risk of acute kidney injury (AKI) compared to trough-guided dosing [8].

The preferred approach to AUC-guided therapy is to apply a software with integrated population pharmacokinetic (PK) models to interpret measured concentrations using Bayesian estimation, referred to as model-informed precision dosing (MIPD) [6]. This allows the concentration to be interpreted in relation to the dosing history, patient covariate values, and expected unexplained variabilities. Practical advantages include the possibility to estimate AUC from only one measured concentration, the use of flexible sampling times and sampling before steady state to improve timeliness of dose adjustments, and the ability to predict the optimal dose strategy for an individual patient.

The concept of MIPD is not new [9], but the adoption in clinical practice has been slow [10]. Barriers for implementation include the need for manual entry of data from the electronic medical record (EMR) to the dosing software, regulatory hurdles because dosing software is regarded as a medical device, and the need for modelling expertise within the health care system [10, 11]. However, user-friendly and CE marked dosing software tools that comply with European Union general data protection regulation have become available in recent years [12], which provides better opportunities for successful MIPD implementation. Furthermore, the uptake of evidence-based recommendations in routine practice can be facilitated by well-designed interventions and targeted implementation efforts [13]. Reporting the intervention development process is encouraged to increase learning across institutions [14].

### Aim

To bridge the gap between evidence and current practice at a Swedish tertiary hospital, we aimed to develop an intervention, including a standardised MIPD workflow, and an implementation plan for vancomycin AUC-guided dosing.

### Ethics approval

Ethics approval was granted by the Swedish Ethical Review Authority for a retrospective audit of vancomycin TDM (Dnr 2021-06864-01) and observations of dosing and TDM sampling (Dnr 2019-04974 and 2020-06080).

## Method

**Fig. 1:**
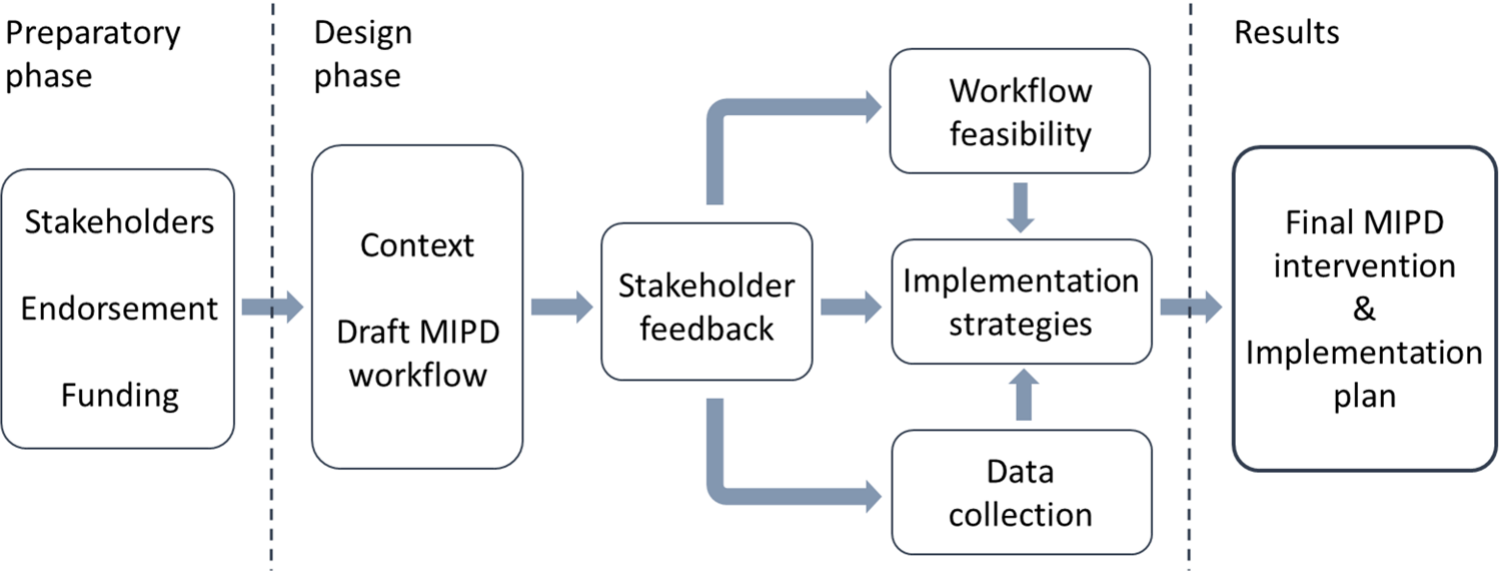
A summary of the development process

The intervention development approach was evidence- and theory-based [15] and guided by the core elements of the UK Medical Research Council’s (MRC) framework for developing and evaluating complex interventions [16]. The process (Fig. 1) included the development of the intervention components, and the parallel consideration of implementation aspects. Guidance for reporting intervention development studies [17, 18] were applied. The work was led by a project team, consisting of five researchers with pharmacy or medical background (Online Resource Table S1).

### Stakeholders, endorsement and funding

In Sweden, health care is decentralised with regional self-government. National authorities and expert organisations provide guidance to the regions, but treatment guidelines may differ across the 21 health care regions. On a national level, the initiative to implement new methods for improved vancomycin dosing was supported and funded by the multisectoral Platform for Innovation of Existing Antibiotics (PLATINEA, www.platinea.se). Further, the project was endorsed by the regional STRAMA group (the Swedish strategic programme against antibiotic resistance, www.strama.se), the hospital antibiotic expert group and the head of department of infectious diseases (ID). Nurses, pharmacists and physicians were identified as key professions for successful MIPD implementation.

### Clinical context

Uppsala University Hospital is an 850 patient-bed tertiary hospital. At the time of study, institutional guidelines advocated vancomycin TDM before the third dose, with a target trough concentration range of 15–20 mg/L in adults [19]. Vancomycin dosing and the timing of TDM were decided at the discretion of the responsible physician. However, an ID physician was commonly consulted for advice on vancomycin treatment, dosing and TDM. Nurses were responsible for TDM sampling and documented times for dose administration and sampling manually in the EMR. Clinical pharmacists worked at selected wards and had an advisory role in prescribing decisions but were not routinely involved in dose decisions following TDM. Vancomycin concentrations were provided 24 h-a-day, 7 days a week and were reported in the EMR with an attached standardised comment on the target range. Dose-prediction software was not routinely in use but expertise in MIPD was available.

### Draft MIPD workflow

A draft workflow for MIPD was developed by the project team to fit the clinical context, with an intention to capture the whole process from TDM initiation to dose decision, and employing a team-based approach. Knowledge about barriers and facilitators [20–24] guided the work. Determinants taken into consideration at this stage related to accessibility of dosing advice, awareness and reminders, communication [21], specialist decision support [21–23] and trust [20, 21], that can be reduced if advice is provided as a remote service not performed at bedside.

### Stakeholder feedback

Representatives from key professions (3 nurses, 6 pharmacists, 2 physicians) (Online Resource Table S1) were selected from wards regularly utilising vancomycin TDM, and based on relevant experience of TDM and local processes. Physicians were selected from the ID department as they frequently give advice on vancomycin dose adjustments. Feedback on the draft workflow was collected during informal discussions with the representatives individually and at two regular staff meetings with nurses and physicians. The discussions focused on opinions on the suggested changes in routines, feasibility of different process steps, training needs and practical problems that might arise. Factors at higher organisational level or legal and economic considerations were not addressed specifically. A member of the project team (MS) took notes of the communicated input, and performed an initial categorisation using the integrated checklist of determinants of practice (TICD checklist) [25]. The categorisation was discussed in a meeting with project team members (MS, EN, AKH), and after a few revisions, consensus was reached (Online Resource Table S2). Design efforts were directed towards the main determinants of practice identified (domains 1, 2, 4), while other determinants were highlighted as remaining uncertainties. The project team concluded that current TDM practices and the potential benefit of MIPD, and quality of documentation in the EMR required further investigation through data collection. In addition, that the feasibility of workflow components involving new processes for data and information transfer needed preliminary testing.

### Data collection

#### Retrospective audit

To assess the quality of current vancomycin TDM practices, a retrospective EMR review (January 2019 to December 2021) was performed at a haematology ward (Table 1). TDM sampling was frequently performed at a time point where many individuals supposedly had not reached steady state (median 16 h, range 8–143 h from treatment start). Trough-based target attainment was low (44%), and in 28% (20/72) of the treatment cycles some degree of AKI occurred [26]. All included patients had other predisposing risk factors for AKI [26], making them especially vulnerable to vancomycin-induced nephrotoxicity. The findings were included in training sessions to motivate the transition to an AUC-guided MIPD approach.

**Table 1:** Retrospective audit of vancomycin TDM practices in 62 vancomycin treated patients at a haematology ward (72 treatment cycles, 305 vancomycin concentrations)

*TDM process*	
Administered loading dose	67% (48/72 treatment cycles)
Median time to first vancomycin sample	16 h (range 8–143 h)
Samples documented as drawn at trough time-point*	83% (253/305 concentrations)
*TDM outcomes*	
Target attainment (15–20 mg/L)	44% (133/305 concentrations)
Above toxicity threshold (20 mg/L)	25% (76/305 concentrations)
AKI (KDIGO stage 1)	14% (10/72 treatment cycles)
AKI (KDIGO stage 2–3)	14% (10/72 treatment cycles)

*Defined as the sample being collected within 30 min before next dose

*AKI* acute kidney injury; *KDIGO* Kidney Disease: Improving Global Outcomes; *TDM* therapeutic drug monitoring

#### Observations

To assess the quality of the documentation in the EMR, observations of dose administration start times [27] as well as TDM sampling times at a haematology (January–February 2022) and an orthopaedic ward (February 2021) were performed. Patients admitted to the wards receiving antibiotics or being monitored with TDM for any medication, were included. An error was defined as the difference between the observed and documented time. The nursing staff was informed about the main aim of the investigation, but not about the focus on documentation quality.

The findings (Online Resource Figure S1) showed that 90% (128/143) of dose administration times were within ± 15 min of documented time [27]. Of the observed TDM sampling times, 81% (85/105) were within ± 15 min. Potential clinical consequences of the errors in terms of false target attainment evaluations were assessed using simulations, and reported elsewhere [27]. In summary, assuming errors of ± 15 min or even ± 30 min would result in an acceptable impact on the target attainment evaluation for vancomycin. However, larger errors did occur occasionally (up to 67 min). Hence, a specific training of nurses focusing on removing large errors in administration time and improve overall quality of sampling time documentation was included as an implementation activity.

### Feasibility of workflow components

Workflow components were tested (March 2020) at the ID ward where all professions included in the workflow were present. Involved staff provided oral or written feedback. Tested components included (1) revised vancomycin TDM orders (pilot test with feedback from nurses and clinical pharmacists), (2) data extraction from the EMR to the software (pilot test with feedback from clinical pharmacists and MIPD consultant), and (3) the dose report templates (read through with feedback from clinical pharmacists and ID physicians). The feedback and revision of workflow components is summarised in Table 2.

**Table 2:** Feasibility testing of selected workflow components

Component	Target profession	Feedback	Intervention refinement
Revised vancomycin TDM orders	Nurses	Time consuming Risk reduced quality Reminders necessary	Old vancomycin TDM orders were kept unchanged
Data extraction from EMR to software	MIPD consultants	Time consuming Extraction at ward level enables follow-up of potential errors	Clinical pharmacists enter dose data into the software
Dose report template	Pharmacists and physicians	Ideally short reports Focus on the AUC and dose advice	Short dose reports to physicians Comprehensive software generated reports documented elsewhere in EMR

*AUC* area under the concentration–time curve; *EMR* electronic medical record; *MIPD* model-informed precision dosing; *TDM* therapeutic drug monitoring

### Implementation strategies

To address determinants of practice, implementation strategies were built into the core intervention as well as in implementation activities. Suitable strategies were derived from the TICD checklist [25] and the intervention mapping (IM) behaviour change techniques [28]. The link to expected outcomes [29] was visualised by a logic model [30].

## Results

### Final MIPD intervention

**Fig. 2:**
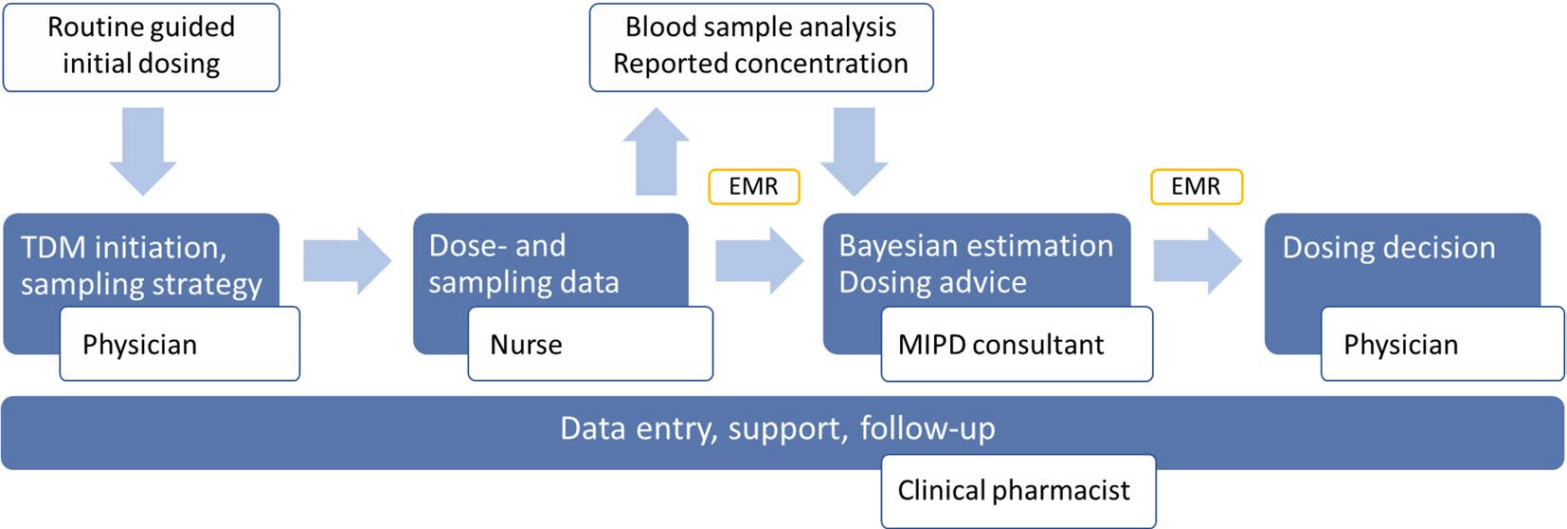
The final intervention workflow for MIPD. The responsible physician prescribes the first doses and initiates TDM as per local routine. Nurses administer the doses, perform TDM sampling and provide accurate data in TDM orders and the EMR. The clinical pharmacist informs the MIPD consultant of a new patient and enters the patient data into the software. Monday-Friday 08:00–16:00 and after the concentration is reported, the MIPD consultant performs the Bayesian estimation and provides a dose report consisting of an estimation of current target attainment and a customised dosing advice linked to the reported drug concentration in the EMR (sign-off required). The MIPD consultant documents the full software generated report in the EMR. The responsible physician is notified of the dose report through an EMR alert, attests the report and considers the advice, makes a dose decision and documents it in the EMR. The clinical pharmacist present at the ward serves as the link between the other roles, reminds the nurses of the sampling routines, discusses complex cases with the MIPD consultant, supports the physicians at TDM initiation and when interpreting dose reports, and assists in patient follow-up. *EMR* electronic medical record; *MIPD* model-informed precision dosing; *TDM* therapeutic drug monitoring

The final intervention consisted of a standardised workflow for MIPD with defined roles and responsibilities as well as processes for data and information transfer (Fig. 2). The workflow included a new role, the MIPD consultant, which can be a pharmacist or physician, with MIPD expertise and an active role in relevant patient care. Furthermore, the responsibilities of the clinical pharmacist were broadened to include MIPD support bedside. Details of subroutines included in the workflow were provided in documents searchable in the hospital document management system and included an instruction on TDM sampling and documentation for nurses, and a dosing software instruction for MIPD consultants and clinical pharmacists (Online Resource Table S3-S4).

Key contextual factors considered as prerequisites for the intervention included the use of EMR, conventional TDM as part of standard care, established ward-based clinical pharmacists, as well as the availability of MIPD expertise and software.

### Implementation plan

The following activities were added to facilitate the clinical implementation of the developed MIPD workflow, here applied for AUC-guided vancomycin dosing.

1. The development of a local clinical routine for vancomycin treatment, written in collaboration with representatives from the involved departments to fit the local context.
2. Staff training:
  • Targeting nurses, a 15-min in-service training provided by a clinical pharmacist. It included a short video enforcing the importance of accurate documentation of dose administration and sampling times for TDM, feedback on current documentation quality derived from the data collection and an introduction to the supportive document on TDM sampling and documentation.
  • Targeting physicians and clinical pharmacists, a 45-min training delivered by a MIPD consultant in conjunction to regular staff meetings. It included the evidence base supporting AUC-guided treatment, the concept and benefits of MIPD, the new process and interpretation of dose reports.
  • MIPD consultants and clinical pharmacists, individual training by an experienced MIPD consultant. It included case-based mentoring, discussion of real-time cases and an introduction to the supportive document with dosing software instructions.
3. Bi-monthly MIPD rounds targeting pharmacists and physicians, led by a MIPD consultant. It provided a forum for updates and exchange of experiences, and an opportunity to achieve a sustained training effect.

### Logic model

**Fig. 3:**
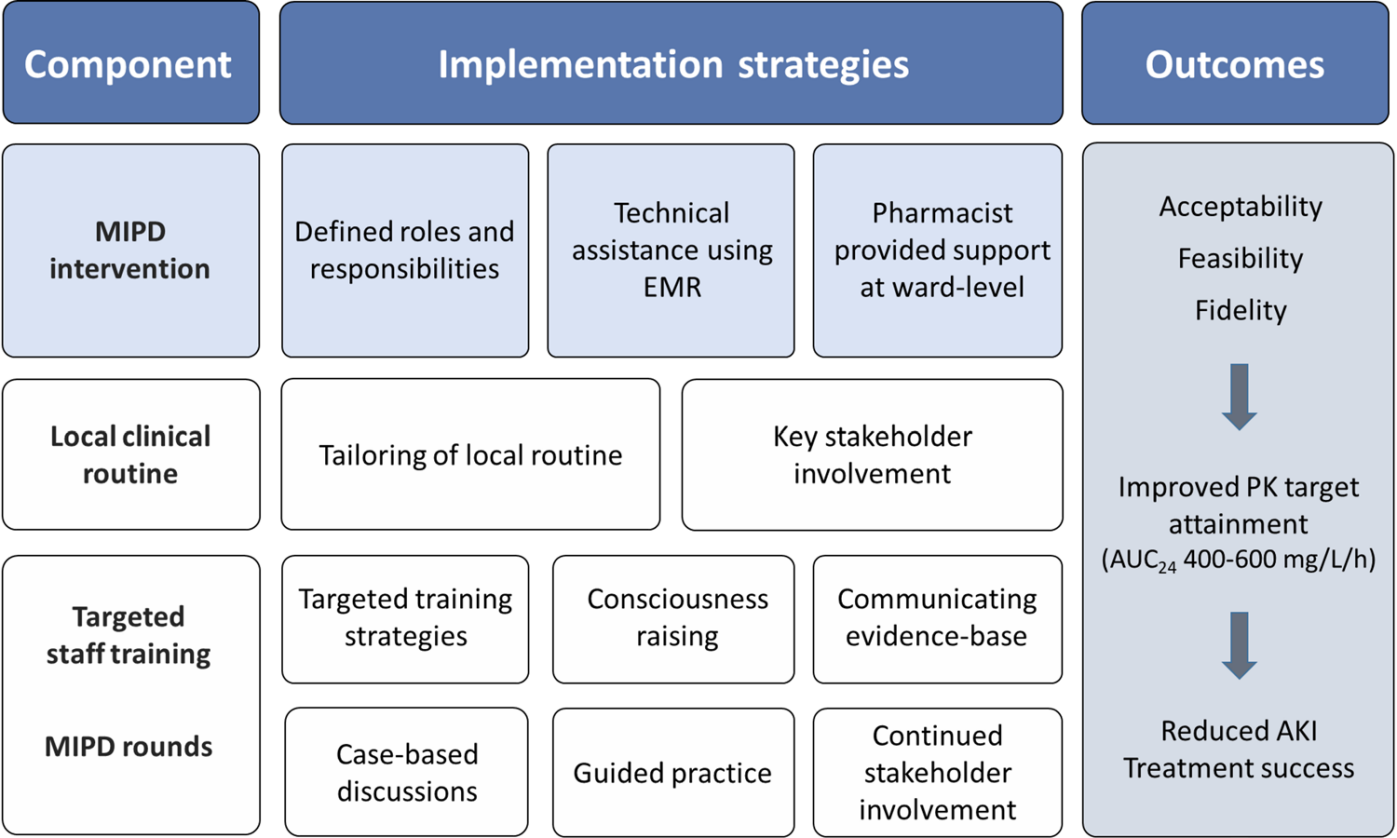
Logic model of the final intervention (workflow for MIPD, including role descriptions and processes for data and information transfer) and the implementation plan specifically oriented towards AUC-guided vancomycin dosing. *AKI* acute kidney injury; *AUC* area under the concentration–time curve; *EMR* electronic medical record; *MIPD* model-informed precision dosing; *PK* pharmacokinetic

The proposed logic model is summarised in Fig. 3, where the components based on selected *implementation strategies* are expected to influence determinants of practice, resulting in expected outcomes as follows.

The first component consisted of the final intervention as described above. By *defining roles and responsibilities*, the determinant team processes was addressed and expected to lead to increased feasibility and fidelity of the intervention. *Technical assistance* in the EMR was used to notify the physician that a dose report is available, improving the determinant referral processes and in the end fidelity. The *collegial support* by the clinical pharmacists to nurses and physicians on the ward provide an interaction to facilitate adherence to the workflow as well as an opportunity for one-on-one *case discussions*. This aimed to improve the determinant team processes as well as physician domain knowledge and was expected to lead to increased acceptability, feasibility and fidelity of the intervention.

The second component was the local clinical routine for vancomycin dosing. The implementation strategy *tailoring* was used to improve the compatibility of the routine with the context, e.g., available dose increments and dose administration routines. Further, the *involvement of key stakeholders* to work with and endorse the routine was used to improve the determinant communication and influence. These strategies were expected to increase acceptability.

The third component included educational activities based on *targeted training strategies*. For nurses, the strategy *consciousness raising* was used to address the determinant knowledge about own practice to improve documentation, enable high-quality dose advice and improve acceptability. For physicians and pharmacists, the strategies *persuasive communication* of evidence and *case-based discussions* were employed to influence determinants domain knowledge, agreement with recommendation and expected outcomes, to improve the acceptability and fidelity of the intervention. For users of the dosing software, the implementation strategy *guided practice* was used to improve skills and the determinant self-efficacy. This was expected to improve fidelity of the intervention. Finally, in the MIPD rounds, the strategies *case-based discussions* and continued *stakeholder involvement* were used to improve the determinants domain knowledge, expected outcome as well as communication and influence. This is expected to improve acceptability and long-term fidelity.

## Discussion

In this study, we developed an intervention consisting of a standardised workflow for MIPD, with defined roles and responsibilities as well as processes for data and information transfer, together with a plan for implementation of AUC-guided vancomycin dosing at a tertiary hospital. We present a structured and pragmatic approach to intervention development in clinical practice, combining published research with local data collection and applying frameworks for theory-informed selection of implementation strategies. The summarising logic model can be used to define research questions for a process evaluation.

Our intention was to introduce MIPD with a team-based approach, from TDM initiation to dose decision. There are publications on MIPD in clinical practice [31–37] including initiatives with similar components as described here, but implemented as an advisory service [31, 32]. In our context, we identified the risk that prescribers would not notify and act upon dose advice communicated in the EMR and/or find it challenging to interpret. Also, a bedside practice and a daily contact with nurses might facilitate the understanding of documented data and feasibility of different dosing regimens. Therefore, we added the role of the clinical pharmacist, serving as the link between the other roles and providing easily accessible support. As users of the software, the clinical pharmacists can use the predicted concentration–time curves to visualise and discuss alternative dosing scenarios, thereby providing learning opportunities within the team. In other institutions, clinical pharmacists are already fully or partially responsible for TDM and vancomycin AUC-guided dosing [24, 33–35]. However, this specific role is rare for clinical pharmacists in Swedish hospitals today, where the main focus is rather on medication reconciliation and reviews as a part of the health care team [38].

Limitations of this study are in part inherent to the applied pragmatic approach. Our identification of determinants of practice was based on informal discussions, rather than interviews with a recording and transcribing process. This reduces transparency and presents a risk of not finding all relevant determinants. However, adopting the suggested full process of developing an intervention [39] would have been lengthy and resource demanding and our process served as a feasible option.

In this study, we present a number of contextual prerequisites for the developed MIPD workflow. This will help others to evaluate the applicability in other settings. By reporting our process and rationale behind selected strategies, we hope that other institutions planning to implement MIPD can adjust the components to their needs. During the development process, the level of detail in the description increased in order for the intervention to be feasible and ready to implement in our setting. Therefore, details about processes for information transfer and instructions in the supportive documents will have to be tailored to fit each context.

The future plan includes a local implementation and evaluation of the fidelity, in terms of quantity and quality of intervention delivery, as well as the feasibility of the intervention. PK target attainment and clinical outcomes will be explored before and after implementation. For large-scale implementation, there are remaining uncertainties. The need for manual entry of data into the software [10] remain a potential barrier and the time consumption needs to be investigated. The integration with the EMR [33, 35] would be a desirable feature. High staff turn-over poses a risk of a waning effect of training initiatives. These should ideally be delivered in a web-based format and included as an introduction of new employees. Furthermore, cost–benefit analyses are warranted, including costs for the MIPD software and workload, the benefit of enabling AUC-guided dosing for vancomycin specifically, as well as the potential to broaden the application to additional drugs. There is a current research focus on developing and applying models for MIPD purposes within a range of therapeutic areas, e.g., oncology, antifungal and immunosuppressive therapy [40–43] as well as novel machine learning approaches [44, 45], and the results of this study can be used to facilitate implementation of these advances in clinical practice. Finally, MIPD expertise within health care is needed for extension to other institutions. A network for interested health professionals and academics could be the next step, where the MIPD rounds included in this study can serve as a model for case-based discussions. This work serves as an important starting point for precision dosing in the clinic, to the benefit of patients.

## Conclusion

An intervention for MIPD with an implementation plan for vancomycin AUC-guided dosing was developed for a tertiary hospital setting. The pragmatic development process can be used as guidance for other institutions with similar context wishing to initiate MIPD.

## Supplementary Information

Below is the link to the electronic supplementary material.

Supplementary file1 (PDF 230 KB)
