# Supplementary material for: Model-informed precision dosing of vancomycin in clinical practice: an intervention development study
Source: Int J Clin Pharm. 2024 Nov 8;47(1):178–86. doi: 10.1007/s11096-024-01822-x (PMC11741990; doi:10.1007/s11096-024-01822-x)
Supplement: Supplementary file 1 — Supplementary file1 (PDF 230 KB) [file 11096_2024_1822_MOESM1_ESM.pdf]

Online resource material

Model-informed precision dosing of vancomycin in clinical practice: an intervention development study

International Journal of Clinical Pharmacy

Maria Swartling, Anna-Karin Hamberg, Mia Furebring, Thomas Tängdén, Elisabet I Nielsen

Corresponding author:
Elisabet I Nielsen, Department of Pharmacy, Uppsala University, Uppsala, Sweden
elisabet.nielsen@farmaci.uu.se

Table S1 Members of the project team, and representatives for key professions for successful MIPD implementation (from ID, orthopaedics, haematology/oncology, clinical pharmacology and regional STRAMA).

	Member/representative
Project team	1. Pharmacist, professor, clinical pharmacy (EN)<br>2. Pharmacist, PhD student, clinical pharmacy (MS)<br>3. Pharmacist, PhD, clinical pharmacology (AKH)<br><br>4. Physician, associate professor, ID (TT)<br>5. Physician, PhD, ID (MF)
Key profession representatives	1. Head nurse (senior)<br>2. Nurse (senior)<br>3. Nurse (senior)<br><br>6. Clinical pharmacist (senior)<br>7. Prescriptionist/ward pharmacist (senior)<br>8. Clinical pharmacist (senior)<br>9. Clinical pharmacist (senior)<br>10. Clinical pharmacist (junior)<br>11. Pharmacist (senior, project team member)<br><br>12. Physician, ID consultant (senior, project team member)<br>13. Physician, ID consultant (senior, project team member)

ID infectious diseases; *MIPD* model-informed precision dosing; *STRAMA* the Swedish strategic programme against antibiotic resistance

Table S2 Stakeholder feedback on the draft MIPD workflow, categorised as TICD determinants for practice. Informants; N = Nurse, P = Pharmacist, D= Doctor/physician. No determinants identified for domains 3 (Patient factors) and 6 (Capacity for organisational change).

Determinant of practice domain	Determinant of practice
**1. Guideline factors**	**Feasibility**<br>Time consuming for nurses to include additional data in the TDM orders (N)<br>Time consuming with manual data entry into MIPD software (P)<br>Time consuming for pharmacists to “look for” physicians for discussions (P)
	**Compatibility**<br>Need for practical dose advice (available doses, dose intervals) (D, P, N)<br>Routine dose administration times vary depending on ward (N)<br>Routine infusion duration varies depending on ward (N)<br>Risk of missing doses documented in other systems because of transfer, e.g., OR, ICU (P)<br>Impractical with special TDM routines for specific drugs, e.g., vancomycin (N)
**2. Individual health professional factors**	**Knowledge about own practice**<br>Unclear if sampling times in TDM orders and EMR can be trusted (P, D)<br>Unclear if documented administration times can be trusted (P)<br>Unclear if documented infusion durations can be trusted (P, N)<br>Unclear if TDM today is executed according to the assumptions (trough, steady state) (P)
	**Domain knowledge, Agreement with recommendation**<br>Unclear if the same AUC target is applicable in all situations (P)<br>Unclear benefit of MIPD and AUC-guided dosing compared to conventional TDM (D)<br>Risk that responsible physician relies too much on the prediction (P)<br>Risk that responsible physician still uses trough only, if reporting AUC and trough (P)
	**Expected outcome**<br>Unclear how well the software/selected model handle special cases (D)<br>Possibility to identify erroneous input data with MIPD (model fit) (P)
	**Self-efficacy**<br>Need for training when involving clinical pharmacists in MIPD (new task) (P)
**4. Professional interactions**	**Communication and influence**<br>Need for specialist involvement to improve acceptability (ID and local speciality) (D, P)
	**Team processes, Referral processes**<br>Risk that responsible physician misses the dose report (P, D)<br>Risk of using the previous trough-based target for TDM outside office hours (D)<br>Risk missing changes in eGFR and that a new prediction is needed (D, P)<br>Need for traceability, e.g., if reported incident (software generated report) (P)
**5. Incentives and resources**	**Availability of necessary resources**<br>MIPD expertise a bottleneck for large-scale implementation (P)
	**Information system**<br>Need for automatic transfer of clinical data to software if large-scale implementation (P)
	**Continuing education system**<br>Not all staff reached by in-service training (N)<br>Staff turnover reduces the effects of in-service training (P)
**7. Social, political, legal**	**Economic constraints on the health care budget**<br>Future software procurement with increased costs (P)
	**Legislation**<br>Need for CE labelled software complying with GDPR (P)

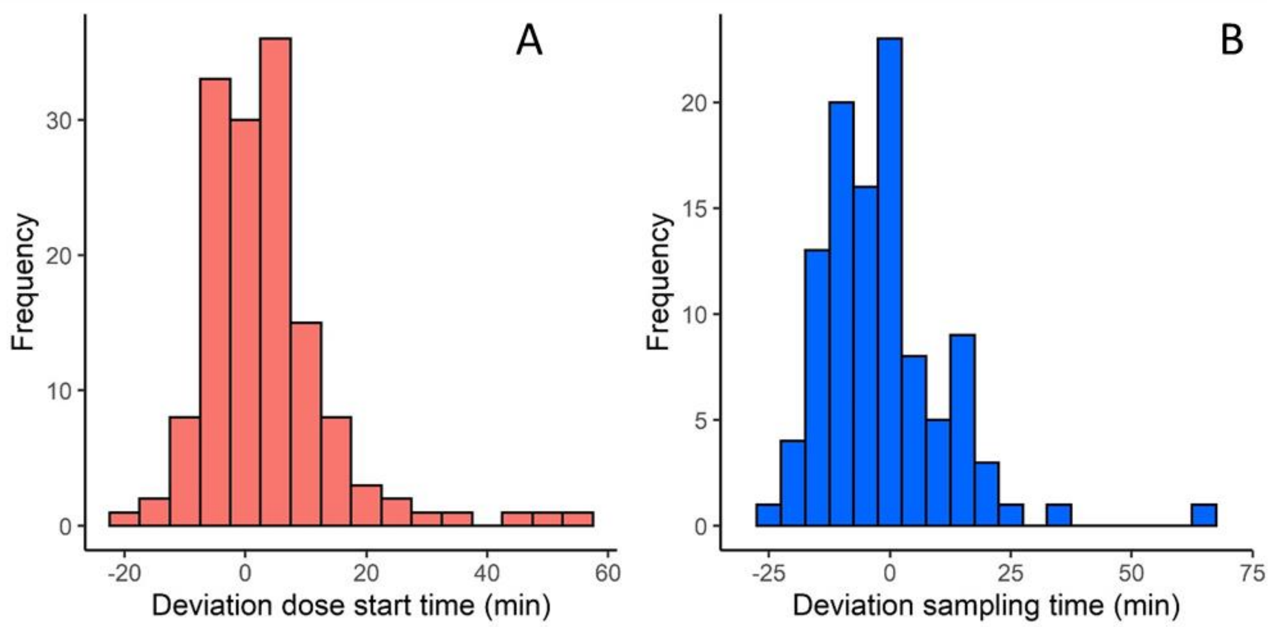

Figure S1 A. The distribution of 143 observed errors in documented dose administration start time at an ICU ward (n=63) and an orthopaedic ward (n=80)[1] (reprinted with permission). Total median 2 min, (range -20-55 min), with 1 min (range -20-55 min) at the ICU and 3 min (range -14-20 min) at the orthopaedics ward. **B.** The distribution of 105 observed errors in documented TDM sampling time at a haematology ward (n=98) and an orthopaedic ward (n=7). Total median -3 min, (range -25-67 min), with -4 min (range -25-33 min) at the haematology ward and 5 min (range -19-67 min) at the orthopaedics ward.
ICU intensive care unit; *TDM* therapeutic drug monitoring

[1] Swartling M, Tängdén T, Lipcsey M, Jönsson S, Nielsen EI. Therapeutic drug monitoring of vancomycin and meropenem: Illustration of the impact of inaccurate information in dose administration time. Int J Antimicrob Agents. 2023;63(1):107032.

Table S3 Summary of subroutines outlined in instructive documents on TDM sampling and documentation for nurses[1]

	**Instructions**
TDM order form	The nurse is responsible for ensuring that the order form contains the correct information. Information that must always be included:<br>o Time of treatment start or dose change<br>o Current dosing regimen (dose and dose interval)<br>o Time of last dose before TDM sampling<br>o Time of TDM sampling<br>If drug administered as an infusion, the following also applies:<br>o The start time of the infusion is stated as the time of the last dose<br>o Standardised infusion rates are used, or deviations noted<br>o If the infusion has to be stopped prematurely or if there are unplanned interruptions in the infusion, this is noted
TDM sampling	The nurse is responsible for TDM sampling and documentation, including:<br>o The exact time for sampling<br>o If the actual sampling time deviates from the planned, the sampling time is changed on the attached test tube label, or by changing the sampling time in the electronic TDM order form<br>When taking samples to determine the concentration of drugs that have been administered intravenously, it is also important to consider the following:<br>o That the catheter or central catheter lumen used for administration of the drug is marked with the substance name. The same lumen must not be used for sampling as drug residues may remain, risking falsely high values<br>o Identify which lumen can be used for drug sampling. If there is no accessible lumen, the sample should be taken peripherally

1. Also available in an instructive video (Swedish): https://uppsala.instructuremedia.com/embed/1d39e502-e3c4-4bcd-9356-49d6ea32d85e

Table S4 Summary of subroutines outlined in instructive document on dosing software (InsightRx, www.insight-rx.com) for MIPD consultants and clinical pharmacists, applied for AUC-guided dosing of vancomycin

**Professional role**	**Instructions**
Clinical pharmacist	o Identify patients treated with vancomycin<br>o Enter patient covariate data (weight, height, S-crea before starting treatment) and dosing information in InsightRx (applies to cases where clinical pharmacists are users of InsightRx)<br>o Inform the MIPD consultant when a new patient starts and stops treatment with vancomycin<br>o Follow up on the reported dosing advice, document reasons for dosing decisions and expected vancomycin trough (to facilitate TDM interpretation during nights and weekends when AUC is not reported)<br>o When adjustment of the dosing advice is needed, use information already entered and checked in InsightRx to evaluate alternative dosage regimens (applies to cases where clinical pharmacists are users of InsightRx)
MIPD consultant	o If no clinical pharmacist is involved, points 1 and 2 above are also included<br>o Add new patients to the code key<br>o Assess reliability in input data, search for test results in lab database (Flexlab) and enter results (S-vancomycin and new S-crea) in InsightRx<br>o Ensure default settings are used in InsightRx, including method for S-crea and eGFR<br>o Select model (primarily Thomson, et al. 2009 for adults)<br>o Perform Bayesian estimation, evaluate model performance (good, intermediate or poor fit) and estimated individual parameters<br>o If intermediate or poor fit:<br>o Check input data<br>o Check if single concentration is deviating (fit info)<br>o If rapid changes in renal function, or unreliable S-crea, and repeated S-vancomycin are available, use "flattened priors"<br>o Based on patient characteristics, evaluate alternative models (e.g., Goti, et al. 2018, Carreno, et al. 2017)<br>o If remaining intermediate fit: Select dosing with expected AUC in the middle of the target range. Communicate that the prediction is uncertain and recommend new S-crea and/or TDM sampling<br>o If remaining poor fit: No dosing advice. Recommend new S-crea and/or TDM sampling<br>o Perform dose predictions using available dose increments (500 mg) and apply preferred dosing interval (8h, 12h, 24h, 48h) when possible<br>o Report and sign the dose report (calculated 24-hour AUC, corresponding trough value and dosing advice from InsightRx as an additional comment in Flexlab (dose report templates[1] are available in Flexlab)<br>o Save the report generated from InsightRx as a pdf and add as an attachment to a daily note from the Academic Laboratory in the patient's EMR

1. Example dose report template in cases where the patient is expected to be at steady state and the AUC is above the target range (dose reduction needed):
   "For an optimal balance between effect and safety, 24-hour AUC at steady state of 400-600 mg*h/L should be reached. At the time of sampling, the calculated 24-hour AUC was XXX mg*h/L (above the target range) with the dose X g every Y hour. To reach the target AUC, the dose should be changed from X g every Y hour to Y g every X hour. This is expected to give an AUC at steady state of XXX mg*h/L, which corresponds to a trough value of about ZZ mg/L. The calculations apply to stable kidney function."
